# Digital Twin Technology In Radiology

**DOI:** 10.1007/s10278-025-01597-1

**Published:** 2025-08-04

**Authors:** Sara Sadat Aghamiri, Rada Amin, Pouria Isavand, Sanaz Vahdati, Atefeh Zeinoddini, Felipe C. Kitamura, Linda Moy, Timothy Kline

**Affiliations:** 1 Decision Neuroscience Laboratory, Center for Brain, Biology, and Behavior, University of Nebraska, Lincoln, NE 68503 USA; 2 Cancer Digital Twin, San Mateo, CA USA; 3 Multiple Sclerosis Research Center, Neuroscience Institute, Tehran University of Medical Sciences, Tehran, Iran; 4 Mayo Clinic Artificial Intelligence Laboratory, Department of Radiology, Mayo Clinic, Rochester, MN USA; 5 Department of Radiology, Massachusetts General Hospital, Boston, MA USA; 6 Department of Diagnostic Imaging, Universidade Federal de São Paulo, Rua Napoleão de Barros 800, São Paulo, SP 04024-000 Brazil; 7 Bunkerhill Health, San Francisco, CA USA; 8 Department of Radiology, New York University Grossman School of Medicine, New York, USA; 9 Department of Radiology, Mayo Clinic, Rochester, MN USA

**Keywords:** Digital twins, Imaging informatics, Radiology, Personalized medicine, Artificial intelligence

## Abstract

A digital twin is a computational model that provides a virtual representation of a specific physical object, system, or process and predicts its behavior at future time points. These simulation models form computational profiles for new diagnosis and prevention models. The digital twin is a concept borrowed from engineering. However, the rapid evolution of this technology has extended its application across various industries. In recent years, digital twins in healthcare have gained significant traction due to their potential to revolutionize medicine and drug development. In the context of radiology, digital twin technology can be applied in various areas, including optimizing medical device design, improving system performance, facilitating personalized medicine, conducting virtual clinical trials, and educating radiology trainees. Also, radiologic image data is a critical source of patient-specific measures that play a role in generating advanced intelligent digital twins. Generating a practical digital twin faces several challenges, including data availability, computational techniques, validation frameworks, and uncertainty quantification, all of which require collaboration among engineers, healthcare providers, and stakeholders. This review focuses on recent trends in digital twin technology and its intersection with radiology by reviewing applications, technological advancements, and challenges that need to be addressed for successful implementation in the field.

## Background

### Conceptual and Practical Definition of a Digital Twin

The digital twin (DT) is a concept originally proposed by Professor Michael Grieves [1]. In 2010, NASA defined a DT as an “integrated multi-physics, multi-scale, probabilistic simulation of a vehicle or system that uses the best available physical models, sensor updates, fleet history, etc., to mirror the life of its flying twin” [2]. Although the DT concept originated in aerospace modeling, it is experiencing a shape-shifting in both its applications and development methodologies [3–5]. In 2020, the Digital Twin Consortium redefined a DT as “a virtual representation of real-world entities and processes, synchronized at a specified frequency and fidelity”. The Digital Twin Consortium, founded by the Object Management Group, is a global alliance of industry, government, and academia working together to unify efforts in standardizing and advancing DT technology across sectors. This consortium serves as a global community focused on establishing best practices for DT applications and shaping the development of emerging standards in the field [6, 7]. While DTs have been widely used in engineering for decades, recent developments have extended their application to medicine and healthcare, including radiology [8–13].

In medicine, DTs can refer to virtual representations of real-world entities such as a patient, magnetic resonance imaging (MRI) scanner, hospital, and drug development process, which enable optimization, monitoring, analysis, and decision-making. The DT technology in radiology can be applied across various domains, including precision medicine, virtual clinical trials, education of radiologists, optimizing medical devices, and improving system performance. In recent years, the number of publications on DTs in healthcare has significantly increased, indicating that researchers have recognized the transformative potential of DTs in medical research [14].

### Evolution of the Digital Twins

Researchers introduced various categories of DTs, reflecting the ongoing evolution of the DT concept. Initially, the DT was conceptualized as a static twin, representing a traditional simulation and modeling, typically involving offline analysis and relying on hypothesis-based mathematical frameworks [15]. As the concept evolved, it introduced the “digital shadow”, which represents a physical object with a one-way data flow from the physical entity to its virtual counterpart, enabling continuous updates to the digital model [16–18]. The most advanced iteration is the “intelligent digital twin”, which incorporates artificial intelligence (AI) to autonomously learn from data, analyze the information, predict outcomes, and optimize the performance of its physical counterpart [18–20].

While the potential of intelligent DT to assist in developing and managing complex systems is recognized, there are concerns about the challenges of generating and utilizing this technology for healthcare purposes. To put DT models into practical use, it is essential to address both technical challenges and ethical considerations.

### Digital Twins and the Healthcare Sector

The market for DT technology has expanded rapidly in recent years. The global DT market is projected to grow to $110 billion by 2028, with a compound annual growth rate of approximately 61%, indicating immense interest in this sector [19]. Major technology firms, including NVIDIA, Microsoft, and Meta, are increasing their investments in DT development [20–22].

This advancement is particularly evident in the healthcare sector, where efforts to implement DT solutions are gaining significant momentum [23]. For instance, Mater Private Hospital partnered with Siemens Healthineers to develop a DT of its radiology department, enabling efficient evaluation of operational changes and significantly reducing the time for improvements [24]. Another example is Quantivly, a company that aims to provide AI-driven radiology operations solutions [25]. Siemens and Atos have collaborated with pharmaceutical companies to represent the DT model of the pharmaceutical industry’s production environment that enables the optimization of the drug manufacturing process [24]. GE Healthcare, in collaboration with Oregon Health & Science University, utilized DT technology during the COVID-19 pandemic to optimize hospital management, enabling statewide tracking and efficient utilization of critical resources [23, 26]. Other use cases include DT for clinical trials, biomarker and drug discovery, surgical planning, device design, biomanufacturing, and hospital management design and care coordination [27]. These examples of DT applications in the healthcare sector demonstrate that this emerging technology requires collaboration among stakeholders, engineers, and healthcare providers.

This article aims to investigate the intersection of DTs and radiology by reviewing applications, technological advancements, and challenges that require to be addressed for their successful implementation in the field. We conducted targeted literature searches using PubMed, IEEE Xplore, Google Scholar, and Scopus, with a primary focus on publications from 2010 to 2024. We prioritized articles relevant to the intersection of DT applications with radiology and healthcare. Also, to reflect current industry trends and technological advancements, we included authoritative white papers, company reports, and official websites from organizations actively developing or implementing DT solutions. These sources were selected based on their relevance, impact, and contribution to the evolving landscape of DT technologies in medicine.

## Applications of Digital Twins

**Fig. 1:**
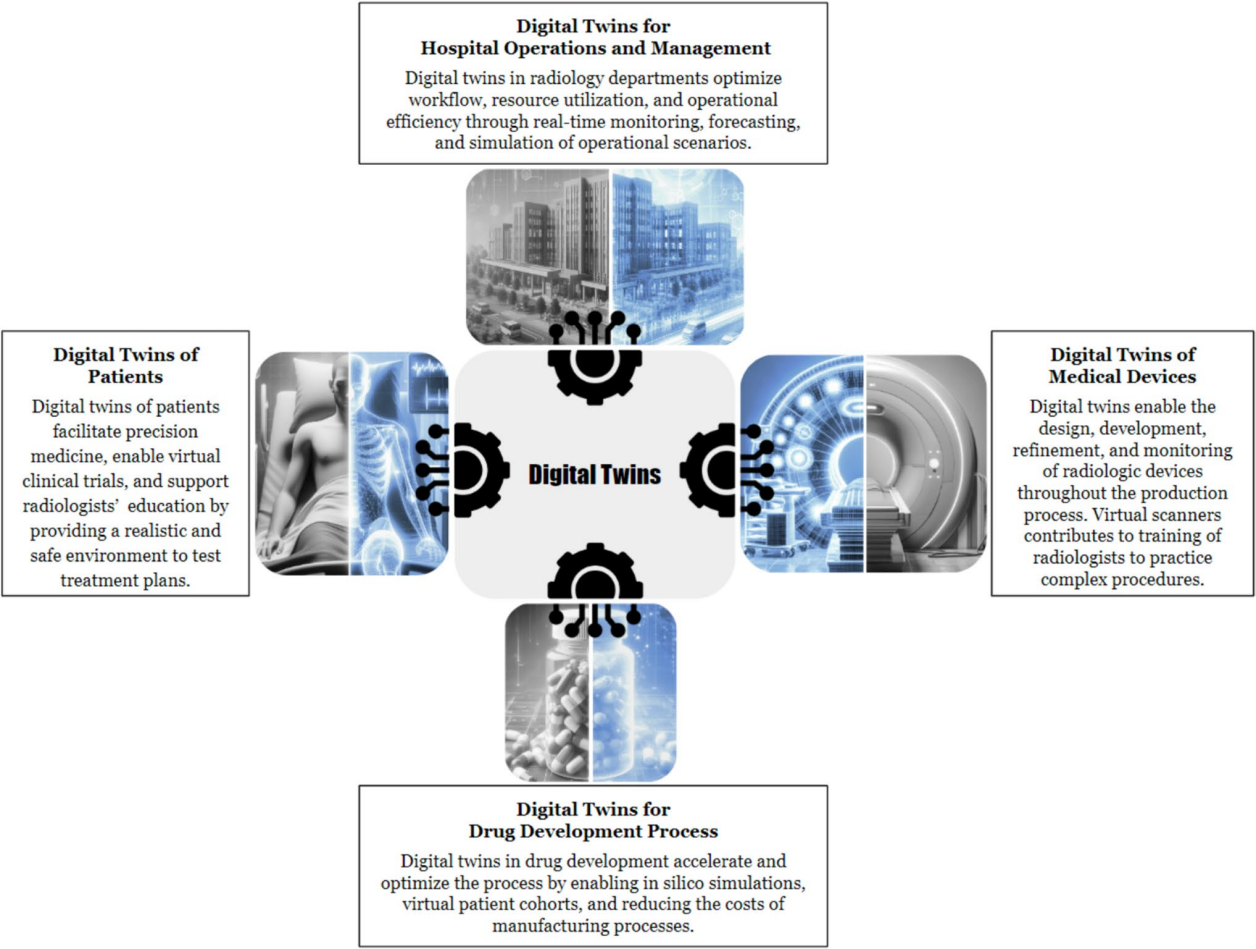
Main applications of the digital twin technology in medicine and radiology

DT technology has shown impressive performance in healthcare, with medical imaging playing a crucial role in its advancements. The medical imaging modalities applied in DT developments include anatomical and functional imaging [28]. DT technology employs medical image data integrated with other biomedical data modalities for various applications. The main applications of DTs include optimizing medical devices (e.g., DTs of MRI machines), improving system performance, facilitating personalized medicine (e.g., patient-specific DTs), educating radiologists (e.g., practicing on virtual scanners and DTs of patients), and reducing the costs of the drug development process (e.g., conducting virtual clinical trials). Figure 1 presents the applications of DTs in medicine. Table 1 summarizes the dimensions of the DT applications presented in this section with the relevant number of references in parentheses.

**Table 1:** Dimensions of digital twin applications

Dimension (Number of occurrences in parenthesis)					References	
Applications (54)	Medical devices (9)				[21, 30–37]	
	Hospital operations and management (9)				[22, 24–26, 37–41]	
	Precision medicine (15)				[44, 46, 47, 52–54, 61, 62, 64–68, 71, 106]	
	Education (9)				[8, 9, 21, 31, 72–76]	
	Drug development (11)				[79–83, 86–90, 92]	
	Clinical Trials (5)				[93–96]	
	Academia (31)		Collaboration (8)		[8, 9, 30–35, 37, 38, 40, 41, 44, 46, 47, 52–54, 61, 62, 65, 68, 72–74, 76, 82, 86–88, 106]	[26, 67, 75, 79–81, 83, 90]
	Industrial sector (14)				[21, 22, 24, 25, 36, 39, 66, 71, 89, 92–96]	
	Federal agencies (1)				[64]	

### Medical Devices

In the manufacturing industries, DTs enable the design, development, refinement, and monitoring of devices throughout the production process [21, 29–31]. DT of radiology equipment, such as computed tomography (CT) and MRI machines, enables remote, real-time analysis, monitoring, and maintenance. These DTs assist in detecting performance issues in radiology equipment (such as detector calibration and radiofrequency coil issues), testing solutions, and preventing potential problems, which is essential for ensuring continuous care for both healthcare providers and patients.

DTs of medical devices also facilitate the design and prototyping of innovative technologies. For example, permanent-magnet-array (PMA)-based MRI systems offer portability and lower costs but have limitations in field strength and uniformity. Researchers are exploring innovative approaches to address these challenges and improve image quality. Huang et al. presented a DT of a PMA-based MRI system that enables precise modeling and simulation of various components such as magnets, RF coils, and gradient coils. This virtual representation supports the design and refinement of these components by allowing real-time analysis of their interactions and performance. Through iterative optimization and simulation of operational scenarios, DTs facilitate hardware debugging, calibration, and evaluation, streamlining the entire development process. This approach minimizes the need for physical prototypes, accelerates innovation, and enhances the overall efficiency of medical device manufacturing [30].

Another example is Virtual Scanner 2.0, an open-source MRI platform for end-to-end simulation, designed as a DT of real MRI scanners in both hardware and software. This technology includes features such as RF pulse design and simulation, hardware-integrated Bloch simulation considering B0 and B1 fields, and non-Cartesian reconstruction. Virtual Scanner 2.0 users can explore the functionalities with Google Colab notebooks for virtual experiments [31].

While the DT of radiology equipment provides virtual representations of real-world systems, virtual reality (VR) provides an immersive experience. DT-VR integration creates a robust training platform for operators, offering a more realistic and engaging learning environment [32]. For instance, in minimally invasive robotic surgery, DT can automatically adjust virtual fixtures in VR training simulations based on real-time procedural context and the surgeon’s actions [33, 34]. The DT-VR integration aims to reduce the cognitive load and skill level required for novice surgeons to operate robotic surgical systems effectively. Also, remote medical operations empowered by VR, robotics, and DT communication facilitate patient’s access to medical personnel regardless of location [35]. Another example, NVIDIA Omnivers, has DT projects that can model hospital environments, operating rooms, medical instruments, and patient anatomy. NVIDIA Clara Holoscan with Omniverse presents the potential of DTs for surgery, which assist medical device developers in creating AI-enabled solutions [36].

### Hospital Operations and Management

DTs have been used to manage hospital workflows and minimize resource shortages by improving resource utilization efficiency [37]. As an example, GE Healthcare has been using DT technology for hospital management to optimize workflow and care coordination. During the COVID-19 pandemic, GE Healthcare and Oregon Health & Science University, in collaboration with nearly all institutions in the state, applied DT technology, updating data from the hospital’s electronic medical records with specificity to the bed level for both census and ventilators across the state. This collaboration resulted in the effective tracking of critical resources, maximizing utilization, and preventing decision-making in isolation across all hospitals in the entire state [23, 26].

A similar approach can be used to establish a DT in the radiology department. Mater Private Hospital, in partnership with Siemens Healthineers, developed a DT for its radiology department. Through this collaboration, a DT of the current radiology processes was created, enabling a review of existing layouts and the identification of potential improvements in the efficiency of the hospital by reducing the waiting time of patients. Using DT technology, the Mater Private Hospital could evaluate new operational scenarios and assess how changes to layout and operations would impact other areas of the hospital. This partnership demonstrated the potential of DT technology to significantly reduce the time spent on trial and error for improvements that previously took months or even years [38, 39].

Quantivly is another instance of DT for radiology operations. Quantivly develops DTs for radiology operations, modeling department complexities rather than individual patients. These DTs integrate real-time monitoring, forecasting, and a Smart Recommendation Engine to optimize workflows, improve performance metrics, and enhance patient care [25].

### Remote Monitoring and Telehealth

**Fig. 2:**
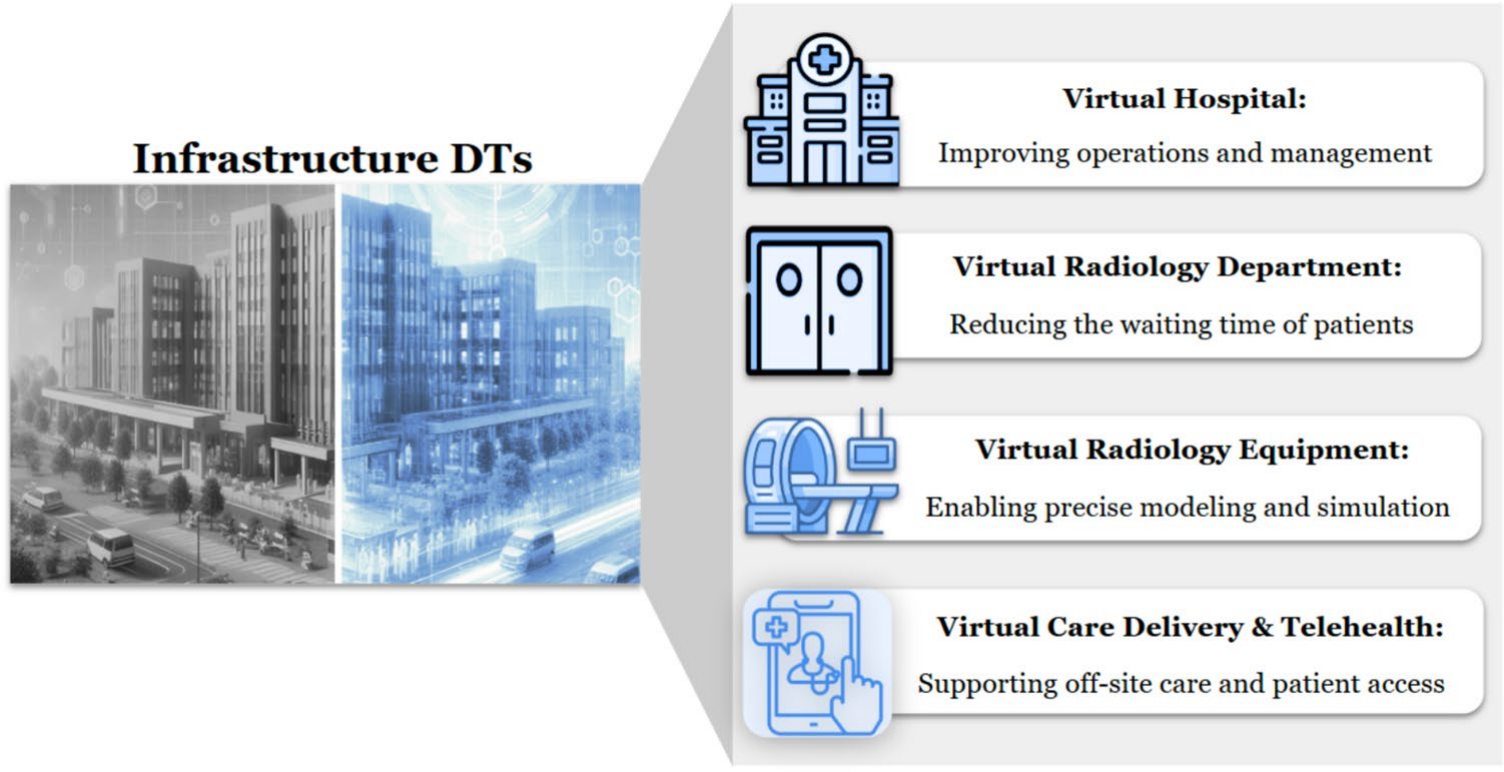
Applications of digital twins as healthcare infrastructure

DT is also in telehealth, such as virtual hospitals, virtual healthcare facilities, and virtual care centers (Fig. 2). A virtual hospital is a healthcare institution that operates entirely in the digital realm without a centralized physical presence, serving as a hub for telehealth services [37]. Medical data from remote e-clinics are transmitted to general practitioners at the virtual hospital, who then diagnose or refer patients to relevant departments. These departments, connected via the Internet, collaborate with specialist consultants globally to deliver continuous remote care, similar to traditional hospitals, but without physical beds, relying on robust interoperability between devices and systems [37].

Another example is DTs of operational control processes assisting in the management of severe trauma cases. To develop a DT of the trauma management process, it is treated as a physical asset represented by the pre-hospital-phase DT (for aid at the accident site) and the operative-phase DT (within the hospital emergency department). The TraumaTracker project is designed for trauma documentation, which is essential for tracking time-dependent acute patient flow and making better decisions to save patients’ lives [40, 41]. Collecting data directly from the accident site assists in developing pre-hospital-phase DT, enabling quicker response times, which are crucial in time-sensitive situations. At the ICU station, the operative-phase DT can detect anomalies sooner and facilitate earlier intervention [41].

### Patient-Specific Digital Twins

**Fig. 3:**
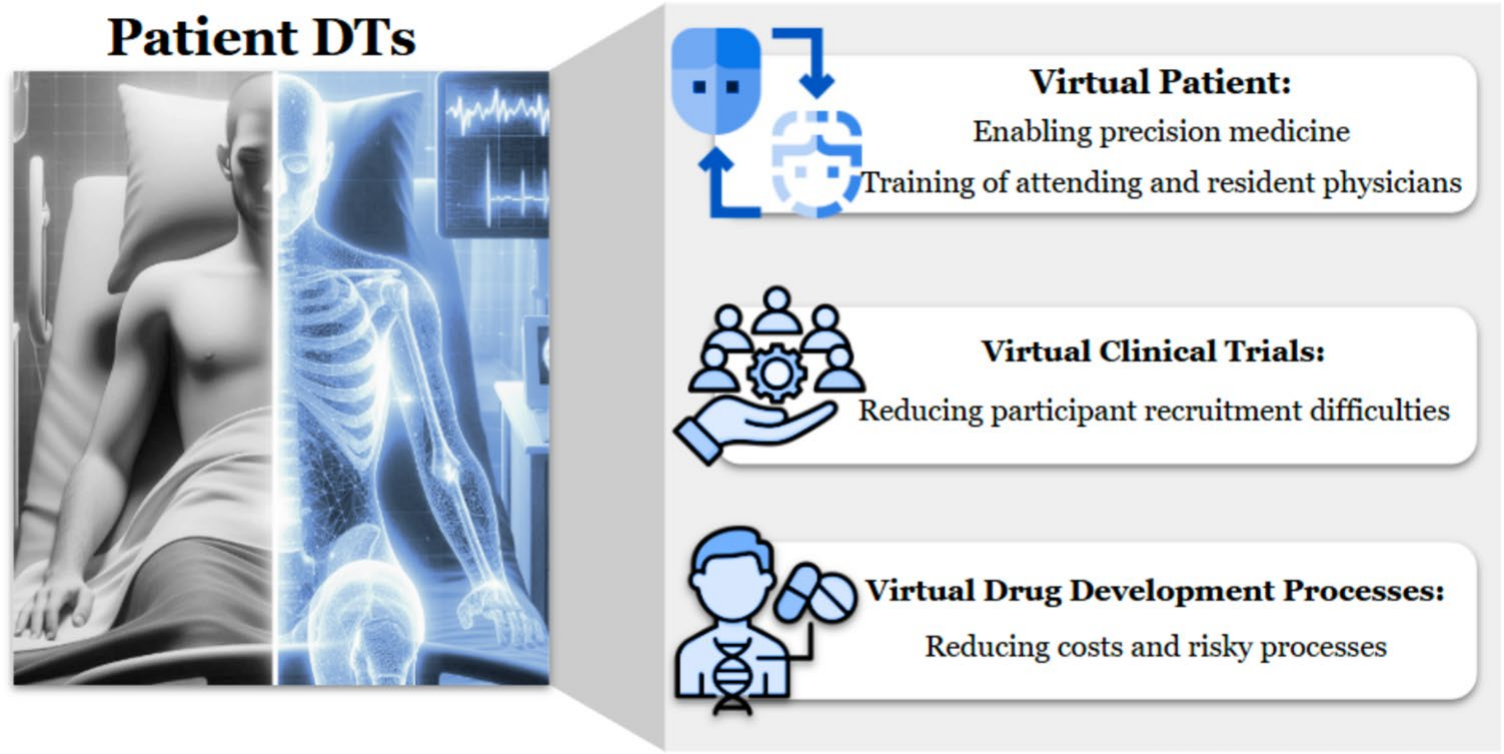
Applications of digital twins as patients

Precision medicine is the optimal disease management using clinical, therapeutic, and diagnostic strategies based on individual variations in a patient’s genetic profile [42] (Fig. 3). Medical imaging plays a critical role in precision medicine, such as screening, early diagnosis, guiding treatment, evaluating response to therapy, and assessing the likelihood of disease recurrence. The success of precision medicine relies on imaging techniques that can accurately classify patients into distinct subgroups who share similar disease characteristics, treatment responses, and prognoses [43]. Advancements in precision medicine require novel approaches, such as DT technology that can integrate medical imaging data with various patient-specific information to customize treatment and prognosis for individual patient needs [44]. Generating a patient-specific DT requires multiscale data, including molecular scale (e.g., blood tests, mass spectrometry, circulating tumor DNA, methylation, genetic sequencing), acellular scale (e.g., extracellular vesicles and their component analysis), cellular scale (e.g., histopathological imaging), organ scale (e.g., functional tests, MRI, and PET imaging), electronic health records (EHRs), family history data, and societal data (e.g., geographical data, economic toxicity analysis, diversity).

Developing multiscale and multimodal DTs of patients can significantly improve patients’ care in various domains such as theranostics, precision oncology, and drug delivery systems. For instance, theranostic DTs enable personalized radiopharmaceutical therapies. Theranostics is a discipline that integrates diagnostic nuclear medicine imaging with radionuclide therapy, using the same target for both molecular imaging and molecular-targeted radiopharmaceutical therapies [45–47]. The optimal selection of imaging modalities in theranostic applications is contingent upon the specific disease, clinical context, and treatment goals. Common imaging techniques utilized in theranostic settings include PET, SPECT, PET/MRI, and PET/CT [48, 49].

Radiopharmaceutical therapy currently faces limitations in achieving optimal treatment outcomes due to tumor characteristics and normal tissue factors [50]. Patient-specific factors, such as tumor microenvironment, tumor heterogeneity, and radiosensitivity, leading to suboptimal dosimetry, increased side effects, and treatment failure, necessitate personalized treatment plans [50, 51].

In radiopharmaceutical therapy, DT technology has the potential to improve treatment efficacy, minimize radiation toxicity, and ultimately enhance patient outcomes [52, 53]. Theranostic DT is an emerging methodology that assists in the design and optimization of radiopharmaceutical therapies by simulating the pharmacokinetic and pharmacodynamic behavior of radiopharmaceuticals within a virtual environment. DTs integrate imaging data, clinical parameters, and patient-specific biomarkers to predict the distribution and effects of radiopharmaceuticals. This personalized approach allows for the optimization of injected activity, treatment schedule, and dose fractionation [48, 52–54].

Another example of the DT function in personalized medicine is the DTs of cancer patients. Precision oncology is an emerging approach that selects cancer treatments based on the unique genetic profile of an individual patient’s tumor, leading to more targeted and potentially effective therapies [55]. Oncology has one of the lowest odds of drug approval at about 5.1%, and it is commonly assumed that less than 5% of adult cancer patients participate in clinical trials [56–58]. Currently, prognosis and treatment decisions in oncology rely on population-based data, which may not account for each individual patient’s variability [59].

In precision oncology, medical imaging is essential for a broad spectrum of indications, ranging from early detection of malignant lesions to response assessment in advanced metastatic disease. As an example, differentiating solitary brain metastasis from glioblastoma multiforme can be difficult and completely change a patient’s management. Another example is the use of Gallium-68 Prostate-Specific Membrane Antigen PET/CT, which could enhance the detection of distant lymph node metastasis and guide clinical decision-making [60]. Radiologic images play a significant role in providing observational data for generating a practical cancer patient DT. DT technology can complement traditional tumor boards and enable a more personalized approach by modeling the unique disease progression of each patient [61, 62]. Cancer patient DT creates a virtual representation of the patient’s tumor and its behavior, enabling in silico exploration of multiple treatment scenarios and allowing for personalized optimization of therapy combinations, dosing, sequencing, and timing [61, 63].

The concept of precision oncology using cancer patient DTs continues to gain interest in both the public and private sectors. The National Cancer Institute (NCI) has announced the Digital Twins Radiation Oncology (DTRO) administrative supplement opportunity, aimed at supporting collaborative and multidisciplinary research in radiation oncology focused on the development of DTs [64]. This administrative supplement funding opportunity aims to build upon the existing infrastructure in radiation oncology, radiation biology, and data science to promote new high-quality collaborative opportunities that span disciplines and data scales. The DTRO program is designed to enable the development of new cross-correlatives and response measures, ultimately optimizing treatment approaches through the use of cancer patients’ DTs. The NCI’s ultimate goal is that these supplements will eventually provide the tools required to test hypotheses in clinical trials. Another example is Indiana University, which is leading the development of DTs for cancer patients to plan immunotherapy in metastatic melanoma patients [65]. The team used multi-scale models of tumor-immune interactions in melanoma pulmonary metastases to develop personalized cancer patient DT templates, refined by canine data and analyzed by AI, to simulate patient trajectories.

In the private sector, Unlearn.AI offers the DT generator model, powered by Neural Boltzmann Machines, to simulate the health trajectories of new patients by predicting how their health might evolve based on initial health data and patterns learned during training [66]. Another application that can benefit from DT technology is optimizing drug delivery systems such as patient-specific convection-enhanced delivery (CED) of therapeutics [44, 67, 68]. The CED for brain diseases (e.g., Alzheimer’s disease, high-grade tumors, and Parkinson’s disease) involves the direct infusion of drugs into the brain through implanted catheters, allowing therapeutic concentrations to be delivered throughout large brain tissue volumes while minimizing systemic exposure [69]. The success of CED depends on the precise placement of catheters and the infusion rate [70]. Patient-specific DTs can assist CED in modeling physiological properties, the specific drugs delivered, and the design of the catheters used for treatment administration [67, 68]. The iPlan Flow from Brainlab, for instance, is a Food and Drug Administration (FDA)-approved software that utilizes MRI imaging data to provide customizable drug delivery procedures [67, 71].

### Education

DT technology contributes to the training of attending and resident physicians by enabling virtual environments to practice a wide range of medical procedures, improving their skills and decision-making [9, 21, 62, 72, 73]. For instance, virtual scanners, powered by DT technologies, provide interactive, realistic simulations of MRI procedures, enabling radiology trainees to learn and practice in a controlled and safe environment [31, 74]. Shanshan et al. presented an MRI equipment DT platform that involves creating a five-dimensional model to improve teaching and training for medical imaging methods. The multi-dimensional physical data are collected for digital human modeling, and a virtual acquisition and image reconstruction technique is introduced for producing images. Healthcare professionals can benefit from DTs for iterative optimization to simulate the entire MRI operation, from preparation and coil selection to patient positioning, parameter setting, and image processing. DT technology ensures that simulation results align with actual equipment performance, thereby improving customized learning experiences and supporting auxiliary design that leads to accelerating digitalization in radiology education [75].

Another example is related to DT-VR platforms for interventional radiologists. Surgical specialties can utilize DT-VR platforms to gain practical experience, test different techniques, and enhance their proficiency without risk to actual patients. This technology provides immersive training that improves surgical skills, hand–eye coordination, decision-making abilities, and patient safety [9, 72]. DTs enable the creation of VR replicas of organs used before, during, and after medical procedures. Zackoff et al. used VR-DT for large-scale immersive onboarding of healthcare staff to a new clinical space, assessing its tolerability, acceptability, and efficacy [76].

### Drug Development

Traditional drug discovery is a costly, time-consuming, and risky process that involves target identification and validation, followed by preclinical and clinical trials. On average, bringing a new drug to market takes 10–15 years of research, and the actual cost of discovering, developing, and launching would exceed USD 1.5 billion [77, 78].

DT technology assists in different steps of the drug development process [79–82]. At the discovery and development stage, it provides a virtual environment for in silico simulations to test various drug targets and treatment strategies before committing resources to real-world trials [83]. In preclinical research, DTs of in vitro and in vivo experiments facilitate predicting toxicity, optimizing parameters for solid-dosage drugs, reducing laboratory practices’ costs, and enhancing manufacturing speed [84]. DTs can provide a multi-scale framework for identifying novel therapeutic targets through the integration of multimodal biological data, combining organ-level (radiologic images) with molecular-scale (omics) via non-invasive methodologies [85–87]. For example, Lu et al. introduced the application of DT in Alzheimer’s disease drug discovery by integrating multi-omics data with clinical data such as imaging data and EHR to construct multi-level representations, and modeling the disease states [88].

At the clinical research stage, patients’ DTs offer digital cohorts for the placebo/standard-of-care control arms of clinical trials [84, 89]. Generating cohorts of patients’ DT accelerates trial timelines, reduces costs, and facilitates drug development [90]. For instance, Phesi and Unlearn.AI offer using patients’ DTs to improve clinical trials [89, 91]. Another example is Siemens and Atos’ collaboration with the pharmaceutical industry to enhance the pharmaceutical manufacturing process by developing DT models as a virtual copy of certain steps in the production process of the factory. This collaboration helps the pharmaceutical industry in using DT technology for real-time analytics to make a more efficient production environment [92].

### Clinical Trials

A clinical trial is a key step in the drug development process, where research is conducted on human participants to assess the safety and effectiveness of a specific intervention [93]. Clinical trials generally follow a three-phase approach, and researchers submit collected data in these trials to regulatory bodies like the FDA for approval. Traditional clinical trials face high cost, time-consuming, and participant recruitment difficulties [78]. Virtual clinical trials and DTs assist in the limitations of traditional clinical trials [94–96]. Virtual clinical trials use digital health technologies to conduct research remotely, streamlining recruitment, collecting data, and monitoring throughout the trial process [94, 97].

DT technology enables virtual clinical trials (as well as other population-based approaches) by generating cohorts of hundreds of patient-specific DTs [61, 95, 96, 98]. For instance, Phesi offers accelerating clinical development with DT analytics [98]. Phesi in collaboration with Massachusetts General Transplant Center utilized DT technology to construct a virtual clinical trial arm for chronic graft-versus-host disease (cGvHD). cGvHD is a condition where traditional trials face challenges due to disease rarity, ethical concerns, and high costs. Using the Trial Accelerator DT platform, the authors created a cohort of 2042 cGvHD patients from a large database of real-world clinical trial data. This cohort and the obtained standard-of-care arm assessed the efficacy of prednisone (a corticosteroid medicine), finding that the results (e.g., an overall response rate of 52.7% at 6 months) were consistent with existing literature. This study suggests that DT technology can replicate control arms in clinical trials as an alternative to traditional methods [98].

In another example, by the Unlearn.AI team, DT technology was used to enhance the design of clinical trials for Alzheimer’s disease. Nearly 7000 clinical records from placebo arms of Alzheimer’s disease trials and observational studies were used to train the Conditional Restricted Boltzmann Machines that generated patients’ DTs. These DTs presented synthetic records with baseline characteristics matched to real trial subjects and projected their disease progression under standard-of-care with or without a placebo. The projections included clinical measures such as the Clinical Dementia Rating Sum-of-Boxes, Alzheimer’s disease Assessment Scale-Cognitive Subscale (ADAS-Cog11), and Mini-Mental State Examination, as well as lab results, vitals, and biomarkers. DTs’ predictions were evaluated against a held-out dataset, and the results showed consistent alignment with actual data within 95% confidence intervals over 18 months. The example of DTs demonstrates that this technology can assist in clinical trials to increase the statistical power of the trial or reduce the required sample size for achieving the desired power [96].

## Generating Digital Twins

**Fig. 4:**
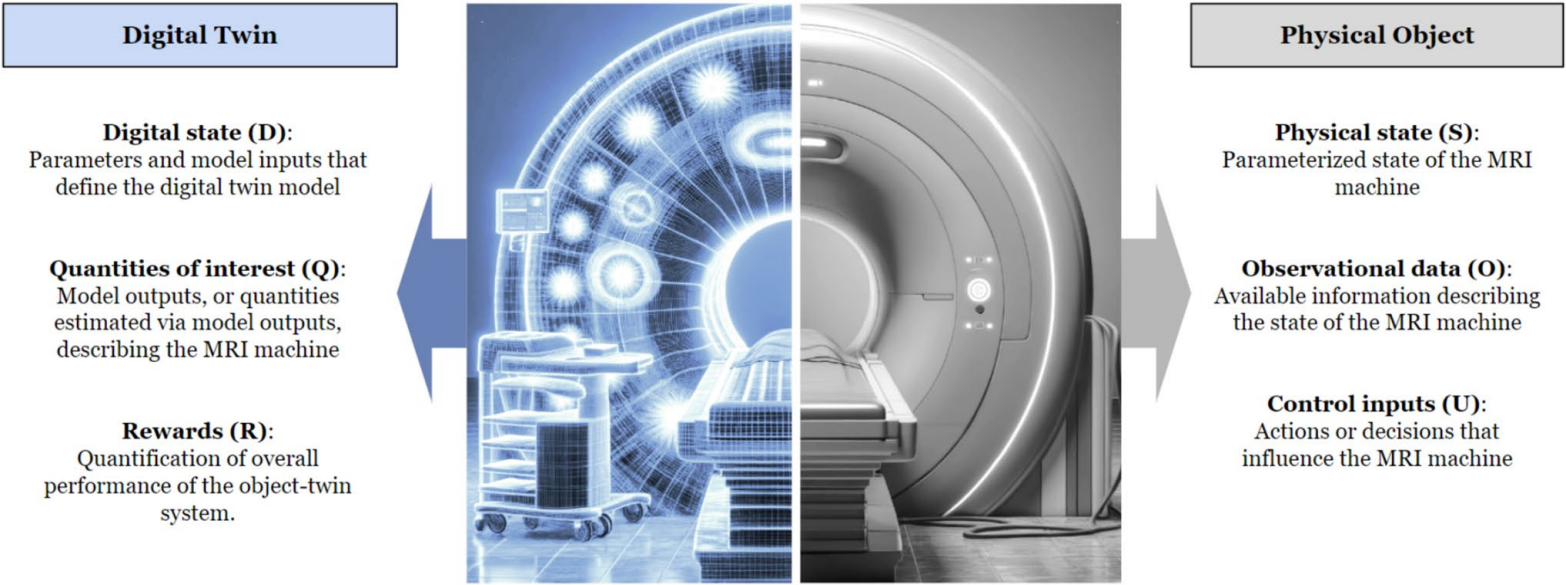
A mathematical abstraction of a digital twin and its associated MRI scanner

A DT is a computational model that provides a virtual representation of a specific physical asset’s structure, behavior, and context, such as a component, system, or process that evolves over time [15, 99]. The mathematical abstraction of an asset–twin system is built upon six major quantities that represent the physical and digital states. Figure 4 shows these key components, including physical state (S), Observational data (O), Control inputs (U), Digital state (D), Quantities of interest (Q), and Rewards (R) [100, 101].

The methods for generating DTs have evolved over time that reflect advancements in computational power and data analysis techniques. DT researchers investigated different methods, including product avatars, digital threads, and digital shadows, which assisted in shaping the technology and eventually led to the development of advanced intelligent DTs [102, 103]. Early approaches proposed digital shadows that represent physical objects with one-way data flow from the physical world to the digital space. For example, Rodríguez-Aguilar et al. described a digital shadow for the health insurance sector [104]. Since digital shadows are limited for real-time feedback loops and bidirectional control, recent advancements in AI suggest intelligent DTs as new approaches for addressing challenges in DT technology [102].

As DT technology has progressed from basic digital shadows to advanced intelligent models, it has been empowered by the ability to integrate diverse biomedical data types. Constructing practical DTs for precision medicine depends on mathematical models designed to integrate different data modalities, including medical imaging, EHR, and personalized omics [105, 106].

Medical imaging provides observational data that can be used as a quantity of the physical state required for generating the patients’ DTs. To accomplish this, radiologic images provide patient-specific measures that play a role in screening, diagnosing, monitoring, staging, evaluating response, and guiding therapy [107]. Personalizing predictive models by radiologic images collected from patients have been demonstrated in several studies [108–110]. Medical imaging enables DT model initialization, while longitudinal updates to patient imaging data also facilitate parameter identification, model validation, and monitoring. The outputs of DTs can also guide the management of data collection for control inputs (e.g. deciding the frequency of follow-up imaging examinations) [111–113].

**Fig. 5:**
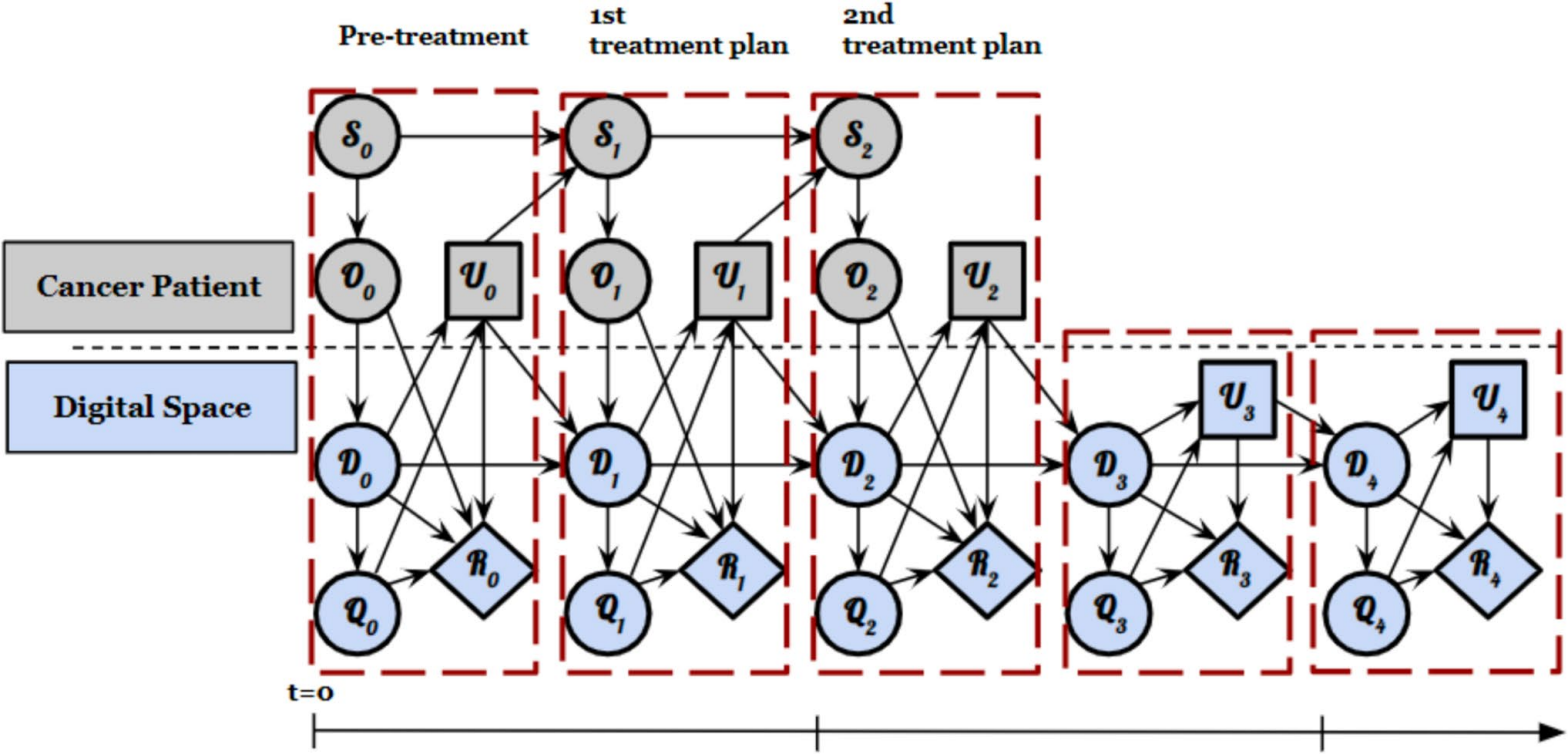
Dynamic decision network of a patient digital twin

Figure 5 presents an example of a dynamic decision network for precision oncology. In the initial step, pre-treatment experiments (U0) are conducted to collect personalized data (O0) (e.g., quantitative imaging) that represent the physical state (S0) which is the cancer patient in this example. Cancer patient DT (D0) utilizes observational data to calibrate the model parameters which enable the predictions of the patient’s behaviors based on quantities for monitoring the patient (Q0) (e.g., tumor shape and cell count, time to progression). The predictions (e.g., tumor response) associated with each potential treatment plan can be quantified as rewards (R0) (e.g., overall survival, risk associated with tumor control, toxicity). The oncologist and patient discuss the potential outcomes and risks, ultimately choosing a treatment plan (U1). Based on the U1, the additional required experiments (O1) are performed to monitor the changes in patients after the first cycles of treatment (S1). The patient undergoes further imaging and clinical exams, providing new observational data to update the DT (D1) and refine the predictions (Q1) and associated rewards (R1). This iterative process continues throughout the course of therapy, allowing for adjustments to the treatment plan (U2, U3, etc.) and ultimately leading to an optimized outcome for the individual patient.

Although radiologic images are one of the critical data modalities in medicine, creating DTs of complex biological systems requires multimodal patient data, incorporating both a large number of variables and their intricate relationships. For example, generating a cancer patient DT requires data and models from the micro level (e.g., genomics), through the meso level (e.g., proteomics and cell behaviors), to the macro level (organ function) [64, 65]. Various modalities assist in personalizing models, such as EHRs, genomics, epigenomics, pathology reports, and environmental data. Data acquisition for establishing a practical DT is a goal-oriented task that provides observational data based on the selected control inputs and rewards. Data integration, modeling, and simulations are among the main challenges in developing DTs. However, the advent of AI has revolutionized DT development, offering a paradigm shift in their capabilities.

**Fig. 6:**
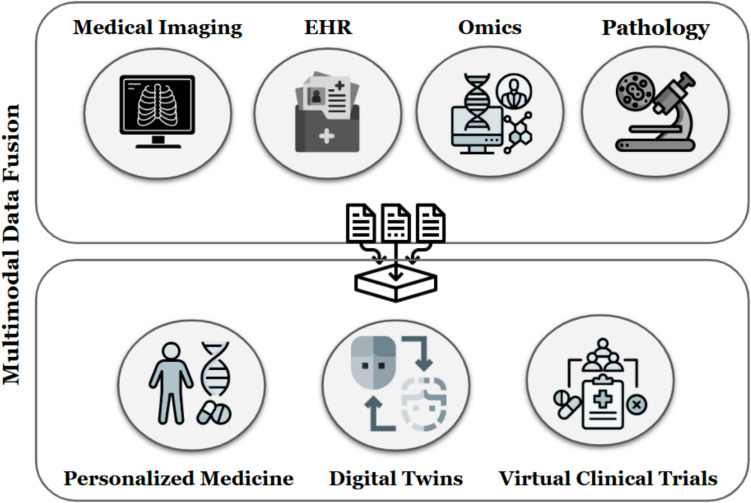
Data modalities and opportunities for multimodal data fusion

In recent years, using AI-generated DTs has been proposed in various application domains as a cutting-edge predictive modeling framework [114–116]. In healthcare, AI-generated DTs have been identified as a promising tool for personalized medicine and drug target discovery [117]. For instance, Díaz et al. presented DTCoach, which provided patient-centered digital coaching during the COVID-19 pandemic [118]. AI-generated DTs, specifically, the role of multimodal AI in integrating data from multiple sources to generate DT models, have been investigated in precision medicine (Fig. 6) [63, 119]. Multimodal AI is a field of AI that focuses on integrating multiple data types from different sources and modalities [120]. Medicine is inherently multimodal, with data modalities spanning EHRs, medical imaging, omics, and more [121]. Med-PaLM Multimodal, for example, is a generalist biomedical AI system, introduced by Google DeepMind [122].

In the context of radiology, Yildirim et al. investigated the potential utility and design requirements for using vision-language model capabilities [123]. Due to the complexities and multi-scale nature of the human body, generating patient-specific DTs involves multimodal data fusion methods to account for a vast number of dynamic impacting factors and sophisticated causal relations [63, 106]. Walsh et al. presented the AI-generated DT that integrates diverse sets of clinical data to enhance clinical trials for multiple sclerosis [124]. Another example by Masison et al. proposed an open-source modular software for developing medical DT models [125].

Building accurate DTs requires extensive, high-quality data, which is often limited in healthcare due to privacy concerns and data silos [14]. On the other hand, medical data collecting is time-consuming, expensive, and encompasses privacy challenges [126]. Another application of AI-generated DTs is creating synthetic data to address privacy concerns and enhance data diversity and quality [14, 83].

Generative AI is a form of artificial intelligence that learns to generate new data from training data [127, 128]. The generative AI models, trained on vast amounts of data, can learn intricate patterns and relationships within patient data, enabling them to generate statistically indistinguishable predicted trajectories of a patient’s condition [14, 126]. By providing synthetic observational data, generative AI facilitates rapid and precise prototyping of intervention strategies within the DT framework [14]. For instance, AI-generated DTs utilizing generative AI assist in drug discovery and clinical trials [83, 117, 129]. Unlearn.AI presented a generative AI-driven DT (providing baseline characteristics matched to those of actual trial subjects) to predict the progression of Alzheimer’s disease [96]. Deeplife offers a commercial solution incorporating AI-generated DTs to simulate drug response by using multi-omics data and underpinning pathway analysis [130, 131].

Another example by Alam et al. described a model architecture to generate DTs of individual patients across multiple disease indications [132]. The examples of AI-generated DTs suggest significant promise for accelerating the development of precision medicine, reducing the cost and duration of clinical trials, and ultimately improving patient care. Integrating AI methods with DTs assist in identifying prognostic factors that increase statistical power, simulating patient trajectories, and streamlining the evaluation of new treatments. This technology has a range of applications, including predicting individual patient outcomes, enhancing the precision of treatment effect estimations, modeling virtual clinical trials, optimizing trial design, and better understanding complex diseases.

## Discussion

DT technology presents a transformative opportunity in radiology, offering various applications ranging from optimizing medical devices to facilitating precision medicine. The evolution of DT technology demonstrates its potential to address critical challenges in healthcare. We suggest that providing a practical DT of a patient relies on the ability to develop multi-scale models encompassing the molecular, cellular, tissue, and organ scales. While modeling at the tissue and organ scales can be informed by radiologic imaging modalities, such as MRI, CT, and PET, the integration of other biomedical data modalities (e.g., genomics, epigenomics, and pathology data) to generate a multi-scale DT for predicting disease progression and treatment outcomes appears to be less developed compared to other DT applications. Given the importance of radiologic images in precision medicine and their reciprocal relationship with DTs, we suggest that radiology can play a significant role in shaping the advancement of DTs in healthcare, while DT technology, in turn, enhances the capabilities and precision of radiological practices.

Our focus in this narrative review was to present various aspects of the intersection between radiology and DTs by providing several examples, rather than criticizing the existing DT development methodologies available in the market. These examples suggest that the potential benefits of DT technology in radiology are enormous; however, several challenges must be addressed for its successful implementation. These limitations and challenges include data-related issues (acquisition, quality, bias, integration, privacy, data security), computational modeling, validation, and uncertainty quantification [12, 133, 134]. Also, ethics and regulations in healthcare DTs are essential to ensure patient safety, privacy, and trust while enabling responsible innovation.

### Technical Challenges and Implementation Barriers

The first significant challenge in generating DTs for healthcare is the accurate acquisition of data, which requires real-time synchronization and multimodal integration. Since practical DTs rely heavily on high-quality data, inaccuracies or incomplete information can result in unreliable models [133]. However, obtaining comprehensive and high-quality health data is often challenging due to its fragmentation across various healthcare facilities. Also, biomedical data can be noisy and subject to biases from factors such as patient variability and data entry errors. Creating and maintaining DTs that accurately reflect changes in a person’s health or biomedical system requires continuous access to longitudinal data, which is often limited and may contain gaps. Moreover, the process of generating and labeling data, particularly in medical imaging and diagnostic contexts, is labor-intensive and prone to human error, complicating data quality maintenance over time and across sources.

For DTs to function effectively, datasets must be balanced to allow fair comparisons across individuals. However, health data can be biased due to the skewed representation of specific demographics or conditions (e.g., racial or gender biases). Using biased datasets can exacerbate existing biases and result in less effective recommendation systems, potentially leading to healthcare disparities. Therefore, ensuring that DT models are free from biases and do not discriminate against individuals or groups is crucial.

Integrating diverse data sources, such as EHRs, imaging modalities, and genomics, into a unified DT model is challenging. Health data is often stored in various formats and systems, leading to interoperability issues among different data formats and medical coding systems. Addressing these challenges requires establishing standardized data formats and interoperability protocols to enable seamless data exchange and real-time synchronization.

A robust implementation of a DT necessitates accurate real-time predictive feedback, which consequently raises the computational costs. Recent advancements in data analysis and computational modeling have led to the development of hybrid methodologies, including AI-generated DTs, to address challenges in this field. A significant challenge is the high cost and need for up-to-date IT infrastructure, including hardware like Graphics Processing Units (GPUs), to support AI growth. The high computational demands can be addressed by employing high-performance computing (HPC) techniques that utilize parallelization to solve, select, or average the DT models while also considering data uncertainties, model limitations, and the availability of new data [135, 136]. The Open Multi-Processing (OpenMP) parallelizes tasks within each node by leveraging multiple central processing units. Message Passing Interface (MPI) distributes computational tasks across multiple computers in a cluster. Emerging GPU-based computing offers significant parallel processing power by distributing tasks across the processing units within video cards. OpenMed, MPI, and GPU-based are among the HPC techniques employed to accelerate model simulations, optimization, and analysis, enabling the creation and maintenance of highly detailed and dynamic DTs [136].

Once a model is developed and calibrated, its predictive capability must be evaluated through model validation, which involves comparing its predictions against new data to assess its accuracy [137]. To determine the model’s validity, a metric is chosen to calculate the error between the predicted outcomes and the actual data. If the error falls within an acceptable range, the model is considered valid. DT uncertainties in observational data, model selection, and model parameters lead to uncertainties in predicted outcomes [138]. The uncertainties include experimental measurement errors, variations in measurement tools with different spatial and temporal resolutions, and errors. These uncertainties introduced during data processing (e.g., cell counting, image segmentation, and registration), underlying model assumptions, for which, to enhance the reliability of the results, model parameters must be carefully considered during model calibration and when interpreting model predictions [113, 139–141]. A realistic expectation of DT technology performance requires uncertainty quantification, which provides controlled and quantified predictions that ultimately increase the reliability of DT results.

### Ethical and Regulatory Considerations

Healthcare DT implementations must comply with stringent privacy regulations governing patient data. The ethical considerations related to DT for personalized healthcare include, but are not limited to, hypercollection, data ownership, data brokerage, unorthodox use, and epistemic injustice. DTs for precision medicine rely on extensive patient data, including sensitive personal health information, making privacy, and security a critical step [142, 143]. Protecting patient confidentiality while accessing necessary health data presents significant challenges. It is essential to protect this information from unauthorized access, breaches, or misuse [144].

Digital obsolescence and service discontinuation affect users’ ability to access and reuse their data in other health care services. Without robust data management and transfer mechanisms, such disruptions can severely impact the continuity and quality of personalized healthcare. Although data ownership remains a debated concept, data brokerage is widespread in digital health, with sensitive user data often shared without explicit consent. This lack of transparency and informed consent poses serious privacy risks, especially in the context of health-related information. Unorthodox use and user noncompliance can compromise the accuracy and reliability of DT data, leading to distorted representations of an individual’s health. This poses risks not only to personalized care but also to clinical decision-making if such data are integrated into broader medical systems. Also, reliance on DTs may lead to epistemic injustice by prioritizing algorithmic outputs over patients’ lived experiences, potentially marginalizing their voices and distorting holistic understanding of health [133, 145–147]. To overcome these challenges, robust measures must be implemented in DT systems to protect patient privacy, ensure data encryption, secure storage, and comply with relevant data protection regulations during transmission and storage, thereby preventing unauthorized access and supporting ethical, secure, and resilient DT infrastructures in healthcare [133, 148].

Advancing ethical development and ensuring the safe use of emerging technologies, such as DT, that involve human subjects is a critical yet complex endeavor. The 1979 Belmont Report laid the ethical groundwork for US federal regulations protecting human research participants. By 1991, these principles were codified into the Common Rule, widely adopted across federal agencies and still in use today [149, 150]. The development of emerging technologies that rely on studying human subjects, such as through clinical trials and the use of patient data, is often necessary for advancing healthcare innovation. The Belmont Report and its resulting regulations establish the ethical foundation for such efforts, emphasizing respect for persons, beneficence, and justice to ensure the responsible and ethical use of patient information in research and development. These principles apply broadly across both research and clinical domains, ensuring that the rights, autonomy, and welfare of individuals are safeguarded throughout the development and implementation of DT applications in healthcare.

We suggest that depending on the DT’s application, different regulations may need to be adopted. For example, the Health Insurance Portability and Accountability Act (HIPAA) and General Data Protection Regulation (GDPR) address protection of personal health information through stringent de-identification protocols and secure transmission standards [151, 152]. HIPAA compliance is further enhanced by the Health Information Technology for Economic and Clinical Health (HITECH) Act, which strengthens data security requirements for electronic health records that might interface with DTs, mandating robust encryption and breach notification protocols [153].

DTs functioning as or alongside medical devices might relate to several regulatory frameworks including the FDA’s Medical Device Regulations (21 CFR Parts 800–1299), Medical Device Regulation (MDR) (EU 2017/745), and the International Medical Device Regulators Forum (IMDRF) SaMD Framework globally [154–156]. Also, the 21 st Century Cures Act provides regulatory pathways for innovative medical technologies [157].

DTs used in research involving human subjects must adhere to established ethical frameworks according to Institutional Review Board (IRB) that ensures protection of patient consent, privacy, and safety during data collection and use [158]. Also in case DTs are employed in clinical trials, Good Clinical Practice (GCP) Guidelines govern their use to maintain data integrity and patient safety [159]. Another example that might apply for DTs is the Food and Drug Administration Amendments Act (FDAAA) that regulates post-market surveillance and adverse event reporting for DT technologies integrated into clinical workflows [160].

Utilizing DT for interpreting laboratory biomarkers or simulating lab results for diagnostic purposes requires consideration of Clinical Laboratory Improvement Amendments (CLIA) standards, ensuring data accuracy and reliability [161]. Another example is the Medical Device Data Systems (MDDS) Guidance that can apply to DTs transferring device data without modification, maintaining data integrity across systems [162]. Given the extensive connectivity requirements of DT platforms, cybersecurity frameworks such as National Institute of Standards and Technology (NIST) provide essential guidelines for protecting against cyber threats and unauthorized access [163]. These standards are crucial for safeguarding sensitive patient information and maintaining trust in clinical DT applications. We suggest that addressing these ethical and regulatory considerations requires collaborative governance models involving stakeholders across healthcare, technology, policy, and patient advocacy domains to establish standards for responsible innovation that prioritizes patient welfare while enabling technological advancement.

## Conclusion

The DT technology within medicine presents a paradigm shift, offering unprecedented opportunities to revolutionize clinical practice, medical research, and healthcare education. This review has explored the evolution of DTs, with a particular focus on their intersection with radiology. From optimizing medical devices and enhancing hospital operations to facilitating precision medicine, conducting virtual clinical trials, and educating radiologists, DT technology is driving innovation in the field. This integration creates a two-directional relationship: while DTs offer substantial advancements in radiology, radiologic images also play a crucial role as a primary data source for generating patient-specific DTs. AI-generated DTs, using multimodal data integration and generative AI, further enhance precision medicine. Despite the advancement in methodology, several limitations remain for the successful implementation of DTs in radiology, including challenges related to data acquisition, quality, bias, integration, privacy, security, computational modeling, and validation. The future direction of DT applications for radiology requires collaboration among stakeholders, engineers, and healthcare providers to overcome the challenges.
